# Matching Psychosocial Support Needs of Parents of a Child with a Chronic Illness to a Feasible Intervention

**DOI:** 10.1007/s10995-020-02925-3

**Published:** 2020-07-01

**Authors:** Miriam Douma, Charlotte P. Bouman, Hedy A. van Oers, Heleen Maurice-Stam, Lotte Haverman, Martha A. Grootenhuis, Linde Scholten

**Affiliations:** 1 Psychosocial Department (G8-136), Emma Children’s Hospital, Amsterdam University Medical Centers, Meibergdreef 9, 1105 AZ Amsterdam, the Netherlands; 2 Princess Máxima Center for Pediatric Oncology, Psychosocial Department, Heidelberglaan 25, 3584 CS Utrecht, the Netherlands

**Keywords:** Parents of children with a chronic illness, Psychosocial problems, Parental psychosocial support needs, Intervention development, Online psychosocial cognitive-behavioral group intervention

## Abstract

**Objectives:**

Parents of children with a chronic illness (CI) are at risk for psychosocial problems. The aim of this study was to refine an existing face-to-face intervention into an online psychosocial group intervention for parents by (1) exploring which themes are important, (2) determine what type of intervention parents would like and (3) assess parents’ practical preferences.

**Methods:**

Parents of children with a CI (0–18 years) were invited to complete an online questionnaire. To acquire more in-depth information, focus groups and telephone interviews were conducted. Descriptive statistics were used.

**Results:**

272 parents (mean age = 43.1 years, 85% female) participated. Three focus groups (15 parents) and seven telephone interviews were conducted. Most important themes were: the CI of the child, family functioning, taking care of yourself, relationships with others and practical support. Parents preferred a group with parents of children in the same age category. At first, parents preferred face-to-face contact. After an explanation and demonstration of an online intervention, parents became more positive about online support, mostly because they could participate from home.

**Conclusions for Practice:**

Parents have a need for psychosocial support focusing on different themes. Professionals should explain and demonstrate an online intervention to parents. Based on these results, *Op Koers Online for parents* was developed. An RCT to assess feasibility and effectiveness of the intervention is currently running.

## Significance

*What is already known on this subject?*: Parents of children with a chronic illness are at risk for developing psychosocial problems.

*What this study adds?*: Based on parental support needs and the themes parents considered as important to address in an intervention, an innovative parent-focused intervention *Op Koers Online for parents* was developed. It is an important contribution to the field, because the focus is on parents themselves, as opposed to existing parental interventions that focus on teaching parents how to support their children. Furthermore, because the intervention has a generic approach, parents of children with rare illnesses have the opportunity to participate in a group intervention.

## Objectives

Parents raising a child with a chronic illness (CI; e.g. asthma, diabetes) are predominantly responsible for managing the child’s illness. Parents are confronted with stressors about their child’s health including practical stressors (e.g. managing daily medical routine) and emotional challenges (e.g. worrying) (Cousino and Hazen 2013; Pinquart 2018). Therefore, these parents are at risk for psychosocial problems (van Oers et al. 2014) and elevated levels of distress (Coughlin and Sethares 2017; Haverman et al. 2013), which can have a negative impact on parents’ coping with illness-related stressors, emotional availability for their children and their ability to manage the child’s illness effectively (Cousino and Hazen 2013). Extra attention for these vulnerable parents is necessary, to prevent and/or reduce psychosocial problems and to help them support their children adequately (Pinquart 2013, 2018).

Psychosocial support of parents has gotten increasing attention in studies and clinical practice over the past few years (Bjorquist et al. 2016; Case et al. 2015; Law et al. 2014; Pelentsov et al. 2015). Emotional, informational and peer support interventions for parents themselves are suggested (Akre and Suris 2014; Glenn 2015). A way to support parents is by using cognitive-behavioral therapy (CBT), which focusses on recognizing cognitive distortions and on teaching parents how to use active coping skills for illness-related problems (Butler et al. 2006). Several CBT intervention programs are available that involve parents. However, those interventions are often primarily focused on teaching the parents to support their child managing the illness (Eccleston et al. 2015; Morawska et al. 2015).

Studies have shown that sharing experiences with others in a similar situation is associated with a decrease of distress and improvement of mental health for children and parents (Niela-Vilen et al. 2014; Ramchand et al. 2017). CBT interventions can be offered in group format. Little is known about the effectiveness of group CBT interventions for parents, but recent research shows promising results on feasibility and effectiveness of this type of intervention for youth with a CI (Douma et al. 2019; Maurice-Stam et al. 2014; Plante et al. 2001; Ramchand et al., 2017; Scholten et al., 2013). A CBT group intervention could be helpful for parents of children with a CI as well.

Over the past few years, a face-to-face CBT group intervention program called *Op Koers* (in English: *On Track*) was developed in the Emma Children’s Hospital (Amsterdam University Medical Centers) and was proven to be effective in improving psychosocial functioning of the child. Involving parents in the intervention contributed to the persistence of the effects (Scholten et al. 2013). The intervention has different modules for children and adolescents with CI, their parents (Grootenhuis et al. 2009; Last et al. 2007) and siblings. Patients with all kinds of CI and their family members are eligible for the intervention: research shows that even though different diagnoses may have different medical treatments, several of the psychosocial problems are the same (Plante et al. 2001). Besides, a generic approach allows for patients with rare illnesses and their family members to participate in a group intervention. *Op Koers* was designed with a generic approach in mind (Last et al. 2007). The face-to-face parent intervention runs parallel to the child intervention: parents learn what the children learn in order to support their child better in daily life. The goal of *Op Koers* is to prevent and/or reduce psychosocial problems by teaching the use of active coping skills. Sharing emotions and experiences with others in the group is an important part of the intervention. All modules have weekly 90-min sessions, for eight weeks, guided by two psychologists (course leaders).

A face-to-face intervention requires participants to visit the hospital. Logistical problems (e.g. travel time) and practical issues (e.g. time of onset of the sessions) have frequently been identified as barriers for participation in face-to-face interventions (Heath et al. 2018; Pettersson et al. 2009). Online interventions eliminate these logistical barriers (Dever Fitzgerald et al. 2010; Hedman et al. 2012) and practical issues are reduced when participation from home is possible (Duppong-Hurley et al. 2016). Moreover, for some parents is easier to type about difficult topics in an online environment instead of talking about it in real life (Heath et al. 2018). Research shows that outcomes of online interventions are comparable to face-to-face interventions (Andersson et al. 2014). Online interventions will not likely replace face-to-face care completely. However, because of the benefits mentioned above, the need for online interventions continues to grow (Ritterband et al. 2003; Ritterband et al. 2013). To increase participation in *Op Koers*, the adolescent group intervention was translated into an online version (Douma et al. 2018; Maurice-Stam et al. 2014). A pilot study shows promising results on feasibility and preliminary effectiveness (Douma et al. 2019). For parents however, a new online intervention that focuses on the parents themselves was needed.

Patient participation becomes more important in intervention development and improves adherence and patient outcomes (Bate and Robert 2006; Blixen et al. 2018). Therefore, involving parents in the development of an intervention is important. Aiming to refine an already existing face-to-face intervention into a feasible online group intervention for parents, the current study was directed at parental needs, by (1) exploring which themes are important for parents to address in the intervention, (2) determining what type of psychosocial intervention parents would like, and (3) assessing parents’ practical preferences for an online group intervention.

## Methods

### Recruitment and Data Collection

A mixed method approach, both quantitative (questionnaire) and qualitative (focus groups/interviews), was used. The only inclusion criterion was being a parent of a child between the ages of 0 and 18 years with a CI diagnosis according to the following criteria (1) onset between aged 0–18, (2) diagnosis based on medical scientific knowledge, (3) the illness is not (yet) curable, and (4) the illness has been present for at least three months, or at least three episodes have occurred in the last year (van der Lee et al. 2007). More than one parent per family could participate when desired.

In order to recruit parents, from September 2014 to February 2015, 57 patient associations were invited to publish a link to the open access questionnaire on their website, social media and/or in their newsletter. Fourteen patient associations (25%) agreed. In addition, the questionnaire was announced on several websites and social media accounts managed by the psychosocial department of the Emma Children’s Hospital. Hardcopy flyers were spread out in the (outpatient) clinic of this hospital.

Parents who were willing to complete the online questionnaire used the open access link. At the end of the questionnaire, parents indicated whether they were interested in participating in a focus group and if so, left their contact details. Interested parents were called by the researcher to schedule the focus groups. Completed questionnaires were anonymously stored in a (secured) website. The focus groups were recorded and transcribed verbatim. When parents were not able to join the focus groups, the researcher offered a telephone interview. During these interviews, extensive notes were taken.

Approval of the Medical Ethical Committee was obtained to conduct the current psychosocial support needs study. Parents gave informed consent prior to participation in the focus group/interview. The study was conducted in accordance with the COREQ criteria for reporting qualitative research (Tong et al. 2007) and the STROBE checklist for cross-sectional studies.

### Questionnaire

#### Background Characteristics

Background characteristics of participating parents (age, sex, marital status, number of children, prior psychosocial support, need for psychosocial support now or in the future for themselves, their child with a CI and possible siblings) and of their child with a CI (age, sex, CI, presence of a second diagnosis) were collected via parent self-report.

#### Support Needs

Parental support needs were assessed with a support needs questionnaire developed for the present study, consisting of 27 questions, including open and multiple-choice questions. The items were selected based on the experiences of care professionals and researchers. The questions concerned (1) which themes are important to address in an intervention (e.g. “What kind of support would you like? For example with a focus on family functioning or own (emotional) functioning”), (2) what type of psychosocial intervention parents would like (e.g. “What type of intervention would you like? For example individual support from a professional or a group intervention with other parents and a professional, either online or in the hospital”), and (3) practical preferences for an online group intervention (e.g. “Which time of onset of the sessions do you prefer? For example in the morning or in the evening”). We refer to Table 3 for the further content of the questionnaire.

### Focus Groups and Telephone Interviews

The focus groups with parents were held in the Emma Children’s Hospital and led by two researchers using semi-structured interview techniques. The goal of the focus groups and telephone interviews was to acquire more in-depth information in addition to the questionnaire. The same sequence of topics was discussed in each focus group and interview, based on the items/themes from the Support needs questionnaire.

### Data Analyses

SPSS version 24.0 (IBM Corp 2016) was used for all quantitative analyses. To indicate support needs, descriptives and percentages were computed. The transcript verbatim of the focus groups and notes from the telephone interviews were read carefully and linked to the items from the questionnaires, to detect any themes regarding support needs or important aspects of an intervention that were not found with the questionnaire.

### Developing a Psychosocial Group Intervention for Parents

Based on the results of the current support needs study, previous studies and the experiences of *Op Koers* developers and course leaders, a feasible online psychosocial group intervention for parents was developed.

## Results

### Participants

A total of 272 parents (mean age = 43.1 years, SD = 7.3 years, 86% female) completed the support needs questionnaire (Table 1). Most parents were married and/or living together (90%) and had more than one child living in their family (87%). More than half of the parents (55%) has had prior psychosocial support from one or more of the following professionals: psychologist (35%), a (medical) social worker (19%) and/or a child life specialist (10%). The mean age of the children (46% female) was 10.7 years (SD = 5.8 years). Parents reported over 60 different types of CI. Half of the parents (51%) indicated a current or anticipated future need for psychosocial suppor. A majority (68%) thought their child with a CI has a current or anticipated future need for psychosocial support and one-third of the parents (33%) indicated a current or anticipated future need for psychosocial support for siblings. Approximately one-third of the parents (35%) indicated that they would like to have contact with other parents with a chronically ill child for support.

**Table 1:** Background characteristics of parents and their children with a chronic illness (N = 272)

	N	Mean (SD) or %
Characteristics of parents		
Age in years	272	43.1 (7.3)
Sex (Female)^a^	235	86
Married and/or living together	246	90
Number of (step)children living in your family		
1	34	13
2	148	54
3	63	23
> 3	26	10
Prior psychosocial support, yes	150	55
… from a psychologist	94	35
… from a (medical) social worker	51	19
… from a child life specialist	27	10
Characteristics of children		
Age in years	272	10.7 (5.8)
Sex (female)	125	46
Chronic Illnesses (main diagnosis)		
Epilepsy	68	25
Neurofibromatosis Type½	57	21
Diabetes Type 1	36	13
Cystic Fibrosis	21	8
Cancer	12	4
Neurological disease (other than epilepsy)	11	4
Migraine	10	4
Other	51	19
Second diagnosis	91	33

^a^Three females filled out the questionnaire together with their partner (male)

A total of 85 parents (31% of all parents) left their contact details for participation in a focus group, of whom 15 parents (18% of the parents who left contact details) participated in three focus groups. The researcher conducted telephone interviews with seven parents (8%). The other 63 parents (74%) could not participate due to several logistical and practical reasons.

### Questionnaire and Focus Groups/Telephone Interviews

Key findings that emerged from the questionnaire and the focus groups/telephone interviews are reported below, grouped into three sections: themes to address in the intervention, type of psychosocial intervention and practical preferences. Data from the questionnaires and data from the focus groups/interviews are presented separately for each section.

### Which Themes are Important to Address in the Intervention

#### Questionnaire

The parents who indicated a need for psychosocial support for themselves (51% of all parents), reported that they would like a focus on their own (emotional) functioning (76%), on how to support their child in living with a CI (70%) and on family functioning (60%). Other themes suggested in the open question were: how to support the child/adolescent in achieving independence, autonomy and self-esteem, and guidance with special (financial) arrangements and different agencies. (e.g. insurances).

#### Focus Groups/Telephone Interviews

Parents indicated that they needed support in accepting the diagnosis and how to cope with several difficult situations while raising a child with a CI (e.g. coping with different future perspectives). Parents also reported that they needed support concerning the impact of the child’s CI on the family and the partner relationship. Furthermore, parents would like to discuss how to take care of themselves next to all parenting responsibilities. Finally, parents would like a focus on how to communicate with their work/school of the child and on practical information (e.g. financial resources). According to parents, an intervention needs to be solution-focused. Table 2 presents parental support needs and how the needs are categorized into four themes to apply in the intervention.

**Table 2:** Overview of parental support needs, how the needs are categorized into themes and applied in the sessions of *Op Koers Online for parents* including group discussions and homework assignments

What do parents want?	How are the needs applied in *Op Koers Online for parents*?		
Parental needs	Theme^a^	Group discussion^b^	Homework assignments*
Guidance in accepting the diagnosis, coping with a different future perspective, adherence/non-adherence and puberty	The CI of the child (*session 2: *“*The hospital*”)	Accepting the diagnosis, how to support the child, successes and struggles in medical treatment of the child and how to handle difficult situations in the hospital	Reading a story about how to cope with the diagnosis & together with your child, make a list of situations for adherence/non-adherence and discuss how to reach adherence
The impact of a CI on family functioning, partner relationship and keeping balance in dividing attention between siblings	Relationships within the family (*session 3: *“*The family*”)	What is the impact of the CI on the child with CI, siblings, the relationship with your partner? What are successes and struggles in your family?	Talk with siblings about worries they have, make a list of things to do with siblings and your partner and have quality time with them each
Taking care of your own body and mind, paying attention to own emotions	Taking care of yourself besides caregiving tasks (*session 3: *“*Taking care of yourself*”)	What is the impact of the CI on your own life and emotional functioning? How do you take care of yourself?	Practice with the relaxation exercise, take time for yourself, give yourself a compliment daily
Communication with bosses/colleagues and teachers of the child about the impact of the disease, practical information about insurances etc	Relationships with others and practical support (*session 4: *“*Extended family and friends*”)	What kind of support would you like to receive/do you receive? What kind of reactions do you get and how does that feel? What works or could be better in the communication with work/school?	Together with your partner, write down difficult questions/reactions you get from others and think of possible ways to react on that

*Homework assignments should be completed every week before the start of the session (except for the first session)

^a^All themes are linked to a session, however, specific content is determined by parents in every session (what they want to discuss)

^b^CBT techniques are used throughout the whole intervention

### What Type of Psychosocial Intervention Parents Would Like

#### Questionnaire

In Table 3, results on what type of psychosocial intervention parents would like are presented. More than half of the parents would like information on a website. Individual counseling with a therapist, face-to-face in the hospital was attractive to a majority of the parents. Regarding a group intervention, parents preferred a face-to-face setting in the hospital. Almost a quarter would like a group intervention in a secured chat with the same therapist. When asked about an individual e-learning, most parents preferred a website with online assistance of a therapist.

**Table 3:** Parents’ answers on what type of psychosocial intervention they would like and their practical preferences for an online group intervention (N = 272)

Preferences for type of intervention	N	%
Information on a website	148	54
Information in a folder	85	31
Individual counselling of a therapist		
Face-to-face in the hospital	176	65
Online individual counselling (the same therapist in all sessions)	92	34
Via an open chat (openly accessible, each time a different therapist)	16	6
Group intervention with other parents and a therapist		
Face-to-face in the hospital	123	45
Via a secured chat (each time the same therapist)	63	23
Via an open chat (openly accessible, each time a different therapist)	16	6
E-learning		
On a website with online assistance	105	39
On a website without online assistance	70	26
Practical preferences for an online group intervention		
Group composition		
Parents of children in the same age category	179	66
Parents of children aged 0–18	52	19
Parents of children with the same CI	9	3
Other (e.g. matched on parent’s own age, no preference)	32	12
Time of sessions		
Evening	153	56
Morning	51	19
Afternoon	13	5
Flexible	14	5
During the weekend	5	2
Other (e.g. no preference, I don’t know)	36	13

#### Focus Groups/Telephone Interviews

All parents preferred a group intervention where they can share experiences and tips with other parents in a similar situation. As parents want to discuss different stages in the life of their child, intervention groups should be composed based on the (developmental) age of the child instead of the CI. Parents expressed a preference for face-to-face support, however, a combination of face-to-face and online sessions would also be appropriate. Most parents preferred to have the first session face-to-face, followed by online sessions. They thought it was important that the intervention was guided by professionals.

Next to the type of intervention, the discussion about timing of the intervention came up in the focus groups. Parents indicated that they received a lot of information when their child was diagnosed with a CI. They experienced trouble finding their way into psychosocial support and/or contact with other parents. Moreover, parents found it hard to seek for and accept psychosocial support. They considered consulting a psychosocial healthcare specialist as a failure and felt like they had to solve the problems themselves. Parents suggested a standard consultation with a psychosocial healthcare provider (e.g. psychologist, social worker) a few months after the child’s diagnosis and they emphasized that an intervention should be easily accessible.

### Practical Preferences for an Online Group Intervention

#### Questionnaire

A majority of the parents would like to participate in a group of parents of children in the same age category (Table 3). More than half of the parents prefers sessions planned in the evening, almost one-fifth prefers morning sessions.

#### Focus Groups/Telephone Interviews

Parents acknowledged the advantages of an online intervention in terms of logistical and practical factors. They mentioned that the possibility to participate from home is a big advantage because it improves accessibility. However, in the beginning, parents were reluctant about an internet intervention and preferred face-to-face contact. After an explanation of an online intervention and a demonstration of what a chatroom would look like, parents became more enthusiastic. In hindsight, parents told they had preconceptions about an online intervention (e.g. difficult to log on) which appeared to be incorrect.

### Development of an Online Psychosocial Group Intervention for Parents

Based on the findings of the current support needs study, knowledge from former literature and the experiences of *Op Koers* developers and course leaders, an online CBT group intervention for parents was developed: *Op Koers Online for parents*. The intervention consists of six weekly morning or evening sessions of 90 min and one booster session four months after the last session. A fixed group of three to five parents chats under supervision of two psychologists (course leaders; the same psychologists throughout the whole course) in a secured chatroom. Intentionally, no webcam for video interaction is used, to keep the intervention anonymous and the threshold for participation as low as possible.

The support needs of parents reported above could be categorized into four main themes for the intervention (Table 2): (1) the CI of the child, (2) relationships within the family, (3) taking care of yourself besides all caregiving tasks and (4) relationships with others and practical support (e.g. school, work). Corresponding topics for group discussion were added. Specific content of each session is determined by the participating parents: what they want to discuss about that theme. The first session is used for introductions and explanation about the intervention. In the last session, time is left to repeat topics, to address matters that have not been discussed due to lack of time and to reflect on the intervention. In addition to the chat sessions, parents can log on to their own personal environment where they can submit weekly homework assignments and view supplementary background information (Table 2). CBT techniques are used throughout the whole intervention. For example, parents learn how to replace negative thoughts by useful, more positive ones (cognitive restructuring) and parents are supported in accepting the diagnosis by focusing on what the parent, the child with CI and the family still can do, instead of what they cannot do because of the CI. Moreover, during the whole intervention, course leaders emphasize the way thoughts influence how people feel and act.

## Discussion

The aim of the current study was to develop an intervention for parents of children with a CI based on their support needs. Support needs were assessed by combining the results from both quantitative and qualitative research.

First, important themes to address in an intervention were explored. A majority of the parents with a current or future need for psychosocial support would like an intervention that focuses on their own (emotional) functioning, how to support their child in living with a CI, family functioning, taking care of themselves, relationships with others (outside the family) and/or practical support. These themes are in line with important themes found in previous research (Case et al. 2015; Paterson et al. 2013; Pelentsov et al. 2015).

The second aim was to determine which type of psychosocial intervention parents would like. The parents in our study preferred a face-to-face intervention over an online intervention. This preference is contradictory to parents’ practical possibilities, which was underlined by the low percentage of parents able to attend the focus group they signed up for. A potential explanation could be that parents have an incomplete understanding of what an online intervention entails. Unfamiliarity with online interventions may lead to negative preconceptions and may cause parents to prefer the more conventional face-to-face setting. In the end, it is expected that practical and logistical advantages of an online format will overweigh parent’s wish for a face-to-face setting. Adequate explanation and demonstration will make an online format more feasible. It should be recognized that an online group intervention requires a well-developed and broadly accessible national IT system. Therefore implementation of *Op Koers Online for parents* was not suitable for use in countries and settings with less access to the internet. Finally, parents indicated trouble finding their way into psychosocial support and/or contact with other parents of a child with a CI. This trouble is corresponding with former research which shows that parents of an ill child can feel isolated, the information they find online is lacking and peer support is desirable (Heath et al. 2018). The suggestion of a standard psychosocial consultation after the child’s diagnosis that parents made is valuable.

The third aim was to assess practical preferences of an online group intervention. Parents preferred to participate in a group with parents of children from the same age category. When composing a group, attention should be paid to the developmental and calendar age of the child. Furthermore, most parents preferred sessions planned in the evening. Some parents indicated not to know their practical preferences for time of onset of the sessions. A possible explanation could be the unfamiliarity with online interventions which can make it difficult to indicate a preference.

Based on the results of the support needs study, an online psychosocial group intervention *Op Koers Online for parents* was developed. Parents’ preferences were mostly met, for example: the themes were established by the parents, the day and time of onset of the sessions is planned in consultation with participants, group composition is based on the age category of the children, a manual to support parents by logging on to the website and entering the chatroom was developed and the intervention is easily accessible (easily accessible website, participation possible from home). However, not all parents’ wishes can be granted. For example, parents preferred one face-to-face session followed by online sessions. However, to ensure anonymity and to eliminate practical and logistical barriers, a face-to-face session was not included. Instead, the course leaders speak to all participants separately on the phone before the first session to introduce themselves, explain the chatroom and answer possible questions of participants. Finally, as revealed from the assessment of parental support needs, there will still be a group of parents who prefer face-to-face over an online setting. For those parents, the option for face-to-face care in the hospital is always available.

Although we tried to involve as many fathers as mothers in the support needs study, the majority of participants were mothers. The underrepresentation of fathers can be due to several reasons, for example that mothers are mostly present at the hospital visits and/or have more time for participation in studies. However, fathers should not be forgotten. The ci of the child puts strain on parental (marital) relationships and both parents are at risk for psychosocial problems. Therefore, *Op Koers Online for parents* was designed for all parents, fathers included. Although the content of the intervention is based the wishes mainly expressed by mothers in the current study, we expect that the intervention will fit to fathers as well. The themes of the sessions are broad and the specific content of the sessions is determined by the participants in the group. When implementing the intervention in clinical practice, clinicians should pay attention to fathers and motivate them to participate.

This study has some limitations. An open recruitment strategy was used, which eliminates the possibility to acquire and discuss information about response rates and differences between non-respondents and respondents. Furthermore, since fathers were underrepresented, we can not comment clearly on the representativeness of the results.

## Conclusions for Practice

Based on parental support needs and the themes parents considered as important to address in an intervention, an innovative parent-focused intervention *Op Koers Online for parents* was developed to use in clinical practice. *Op Koers Online for parents* can be offered to parents after receiving the child’s diagnosis. The intervention is an important contribution to the field, because the focus is on parents themselves, as opposed to existing parental interventions that focus on teaching parents how to support their children. Furthermore, because the intervention has a generic approach, parents of children with rare illnesses have the opportunity to participate in a group intervention. An important finding is the fact that parents are reluctant about the online aspect of the intervention. Caregivers should be aware that it is important to explain and demonstrate the online intervention to parents, and to discuss possible preconceptions and/or misconceptions. For *Op Koers Online for parents*, information booklets and an extensive login manual for (potential) participants were made. Another important finding is that parents suggest a standard psychosocial consultation after receiving the child’s diagnosis, to overcome the barriers for seeking for psychosocial support. We suggest that medical staff direct parents to the psychosocial department of the hospital for a standard consultation when their child is diagnosed with a CI.

Future research should examine the effects of *Op Koers Online for parents*. An RCT to assess feasibility and effectiveness of the intervention is currently running (Douma et al., 2018).
